# 2025: A year of resilience and resourcefulness

**DOI:** 10.1371/journal.pbio.3003582

**Published:** 2025-12-18

**Authors:** Joanna Clarke, on behalf of the PLOS Biology Staff Editors

**Affiliations:** 1 Public Library of Science, San Francisco, California, United States of America; 2 Public Library of Science, Cambridge, United Kingdom

## Abstract

2025 was marked by upheaval and uncertainty for many within the life science community. In this Editorial, we reflect on the past year and highlight some of the many research achievements that give us reasons to be thankful.

## Teaser

2025 was marked by upheaval and uncertainty for many within the life science community. As we reflect on the year that has gone, we highlight some of the many research achievements that give us reasons to be thankful.

2025 will be remembered as a year that sent shockwaves through the scientific community. From the uncertainty caused by federal funding cuts to life sciences research in the USA to the sobering reality of the destruction of laboratory space and disruption to science as a result of armed conflict around the world, I returned from maternity leave part-way through the year to a community that felt more cautious, more considered in their outlook, yet just as passionate about their research. As an editor, I have heard from researchers who have had to scrabble to find funds to keep their labs afloat and from those who are having to fight against rampant misinformation circulating about their research fields. Indeed, for many, 2025 will not be a year to look back on fondly. Yet, despite the challenging circumstances that many researchers around the world have faced this year, we have seen an amazing degree of resilience, and the scientific discoveries have continued apace.

Breakthroughs in organismal physiology and disease-related research mean that we now know more about the genes associated with obsessive-compulsive disorder [1] and the inner workings of mitochondria recycling [2], which is defective in Parkinson’s disease. On a more practical level, a new protective antibody against the malaria parasite was discovered [3], and positive results were reported for the first time for gene therapies for several diseases, most notably for Huntington’s disease, which was able to slow disease progression by 75%. These discoveries are at early stages, but will give hope to many either living with or caring for those with these conditions. At *PLOS Biology*, we have published research this year exploring the potential clinical application of cytosine base editors for mitochondrial DNA gene editing, how homeostatic sleep mechanisms influence brain fluid dynamics, and the effects of aging on the ovarian immune system. We also featured an exciting collection of comment, review, and opinion articles on cancer crosstalk across spatial and temporal scales and a mini-focus on the emerging field of interoception research. Notably, the awarding of the 2025 Nobel Prize in Physiology or Medicine to Mary Brunkow, Fred Ramsdell, and Shimon Sakaguchi for their work on peripheral immune tolerance has recognized the importance of understanding the way the immune system fine tunes its responses to self and external threats, a theme that we have covered in Essays on oligodendrocytes and disease-associated microglia.

The picture in ecology and environmental science has been less rosy, with the failure to reach consensus on phasing out fossil fuels or reducing deforestation at the recent COP30 meeting, and reports of a rapid decline in the number of butterflies in the past 20 years [4], catastrophic levels of coral bleaching [5] and the buildup of microplastics in agricultural soil [6]. The fundamental importance of soil biodiversity is something we have highlighted in a recent Essay, and will be critical to understand for efforts to feed the growing global population. However, the outlook is not completely bleak. We are still discovering amazing creatures that can survive in the harshest environments, such as the bright yellow worms that thrive despite living in toxic levels of sulfur and arsenic, which could offer potential solutions for global challenges related to environmental degradation. Importantly, there are steps that we can all take to address biodiversity loss and look after the planet.

In cell and developmental biology, 2025 saw reports of a new type of organelle (the hemifusome) [7] and a new type of ‘cell cleansing’ process (cathartocytosis) [8], as well as the publication of a spatial map of the developing spinal cord [9] and a stem cell-derived embryo model of primate gastrulation [10]. Research in our pages has revealed the detailed architecture of *Corynebacterium glutamicum*’s highly unusual mycomembrane, shedding light on how mycomembrane-containing species have evolved distinct envelope architectures to thrive in diverse environments. Another paper reported on how to temporarily recreate simple ancestral proto-feather-like structures in chickens, akin to the putative simple feathers that are thought to have first appeared in the common ancestor of dinosaurs and pterosaurs during the Early Triassic period. We have also touched on the thorny problem of de-extinction, following a wave of highly publicized press releases from Colossal Biosciences announcing, among other things, the ‘de-extinction’ of dire wolves.

In cognitive and behavioral sciences, this year we’ve discovered the rules of synaptic plasticity during learning [11] and new ways that ants work together to be ‘superefficient’ [12]. Research in *PLOS Biology* has shown how food-access strategies learned by juvenile great tits are strongly influenced by their broader social environment and how humans process feedback from avatars differently to that from fellow humans. Studying human brain function outside of the lab can be challenging, but as interactions with avatars and computer interfaces become increasingly common in our daily life, it will be important to understand how our brain processes these exchanges.

Within the wider research landscape, there has been a renewed focus on trust and reproducibility in science in 2025, particularly as we think about how best to incorporate artificial intelligence (AI) into the scientific process. Exemplary work in this area has highlighted an explosion in the publication of formulaic articles that report single-factor associations using data from the US NHANES medical database, a phenomenon the authors attribute to AI-assisted papermills. As publishers scramble to keep pace with the rapid developments in AI-assisted research, writing, editing and reviewing, it will be interesting to see what becomes acceptable use within the research community, and what is seen as poor ethical conduct. We are often quick to decry the use of AI in generating artwork (such as the notorious recent case of a paper on autism, where a figure in the paper featured nonsense words), but are seemingly more tolerant of the use of AI to summarize, write or edit text, raising questions of authorship in articles. Where the line will eventually be drawn remains a matter of open debate.

As a difficult year draws to a close, it remains for us to thank the authors, reviewers, and members of our editorial board who have worked tirelessly to help us fulfill our mission to publish the full remit of life sciences research without compromising on quality, and to thank you, our readers, for choosing to support open science. To those academic editors who stood down this year after many years of providing advice, we thank you for your service, and we extend a warm welcome to those who have newly joined our ranks; your contributions will be vital in the years to come. The world in which we practice science may be changing, but our commitment to drive forward open science remains the same.

## Abbreviation:

AI: artificial intelligence
